# Prediction of Subsurface Microcrack Damage Depth Based on Surface Roughness in Diamond Wire Sawing of Monocrystalline Silicon

**DOI:** 10.3390/ma17030553

**Published:** 2024-01-24

**Authors:** Keying Wang, Yufei Gao, Chunfeng Yang

**Affiliations:** 1 Key Laboratory of High Efficiency and Clean Mechanical Manufacture of MOE, School of Mechanical Engineering, Shandong University, Jinan 250061, China; 2 Key Laboratory of Manufacturing Equipment of Shaanxi Province, Xi’an 710048, China

**Keywords:** diamond wire saw, monocrystalline silicon, subsurface microcrack damage depth, surface roughness

## Abstract

In diamond wire saw cutting monocrystalline silicon (mono-Si), the material brittleness removal can cause microcrack damage in the subsurface of the as-sawn silicon wafer, which has a significant impact on the mechanical properties and subsequent processing steps of the wafers. In order to quickly and non-destructively obtain the subsurface microcrack damage depth (SSD) of as-sawn silicon wafers, this paper conducted research on the SSD prediction model for diamond wire saw cutting of mono-Si, and established the relationship between the SSD and the as-sawn surface roughness value (SR) by comprehensively considering the effect of tangential force and the influence of the elastic stress field and residual stress field below the abrasive on the propagation of median cracks. Furthermore, the theoretical relationship model between SR and SSD has been improved by adding a coefficient considering the influence of material ductile regime removal on SR values based on experiments sawing mono-Si along the (111) crystal plane, making the theoretical prediction value of SSD more accurate. The research results indicate that a decrease in wire speed and an increase in feed speed result in an increase in SR and SSD in silicon wafers. There is a non-linear increasing relationship between silicon wafer SSD and SR, with SSD = 21.179 *R*_a_^4/3^. The larger the SR, the deeper the SSD, and the smaller the relative error of SSD between the theoretical predicted and experimental measurements. The research results provide a theoretical and experimental basis for predicting silicon wafer SSD in diamond wire sawing and optimizing the process.

## 1. Introduction

Monocrystalline silicon (mono-Si) is currently widely used in semiconductor chip substrates and photovoltaic solar cells [1,2]. Silicon wafer processing adopts diamond wire saw slicing technology, which has also been widely used in the slicing processing of other hard and brittle materials such as silicon carbide [3], sapphire [4,5], and stone [6]. During the diamond wire saw slicing of mono-Si, the material brittleness removal results in residual microcracks on the sawn wafer subsurface, causing microcrack damage. The SSD significantly affects the quality of silicon wafers, reducing their fracture strength and increasing the breakage probability. In addition, the SSD can affect the subsequent processing time and cost. Therefore, obtaining the SSD generated in mono-Si wafers has a very important guiding role for subsequent processes. Based on the SSD, the processing parameters or processing time for subsequent processes can be formulated.

The experimental detection of SSD in the subsurface of silicon wafers is generally measured using cross-sectional microscopy, which is time-consuming and laborious, and the specimen cannot be used again after measurement. Therefore, it is necessary to obtain a fast and non-destructive SSD prediction method for wire saw machining. In recent years, scholars have been committed to the theoretical modeling of diamond wire saw cutting processes to predict the SSD under determined diamond wire and machining parameters. One method is to mathematically model the diamond wire saw cutting process [7,8,9]. Based on the theory of indentation fracture mechanics, this method proposed an SSD prediction model that comprehensively considered both median and lateral cracks below the abrasive during wire saw cutting, and theoretically predicted the SSD of silicon wafers. In addition to using mathematical modeling methods, the finite element analysis modeling method has also been applied to the analysis of SSD in machining such as grinding [10] and diamond wire saw cutting [3,11,12]. The SSD was analyzed following the application of maximum stress on the wafer, when subsurface cracks occur during slicing processing. The numerous aforementioned studies have constructed the relationship between diamond wire parameters, sawing process parameters, and the SSD of wafers in wire saw cutting, achieving simulations predicting SSD in wire sawing. However, these modeling methods also have certain limitations, such as complex calculation processes, and low calculation efficiency. Therefore, it is desirable to establish a rapid prediction method for the SSD in sawing.

Numerous scholars have found that there is a certain relationship between the SR formed by material brittleness removal and the SSD when processing hard and brittle materials. Scholars mainly focus on grinding and lapping technology in this area, and the materials processed are mostly optical crystals, based on the theory of indentation fracture mechanics. Li et al. [13] conducted an earlier study on the relationship between SSD and SR in grinding optical materials and constructed a theoretical ratio between the two. The established model equates SSD with median crack depth and peak-to-valley surface roughness with lateral crack generation depth. Subsequently, scholars have conducted extensive theoretical and experimental research on the relationship between SR and SSD in the grinding process of materials such as glass ceramics [14,15], optical glass [16,17], and fused silica [18,19]. The research results indicate that by measuring the SR, the SSD can be quickly and accurately predicted. Because the influence of various factors on machining quality can be reflected in SR, this kind of SSD prediction method based on machined SR has more engineering application value and guiding significance.

At present, there is currently limited research on the relationship between SR and SSD during diamond wire saw machining mono-Si, which is the focus of this paper. The relationship between SR and SSD in this paper has been established. During this process, the effect of tangential force and the influence of the elastic stress field and residual stress field below the abrasive on the propagation of median cracks are comprehensively considered. Therefore, the theoretical propagation morphology and length calculation of median cracks are more in line with practical laws. Furthermore, SR and SSD were evaluated using surface contact roughness meters and scanning electron microscopy in a sawing experiment, respectively, and then the relationship between SSD and *R*_a_ was refined. Additionally, the coefficient *η* which considers the influence of material ductile regime removal on SR values has been added, making the theoretical predicted value of SSD closer to the actual value. The research results provide a theoretical and experimental basis for quickly predicting the SSD of diamond wire saw cut silicon wafers and optimizing the cutting process.

## 2. Microcrack System beneath Cutting Abrasive Particles

**Figure 1:**
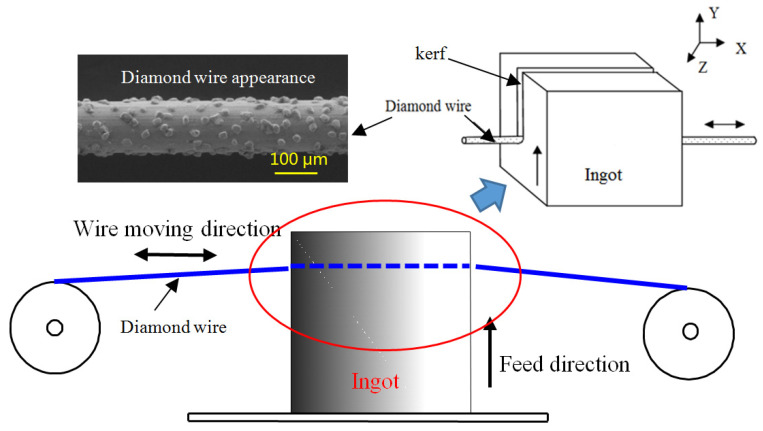
Schematic diagram of diamond wire saw cutting process.

**Figure 2:**
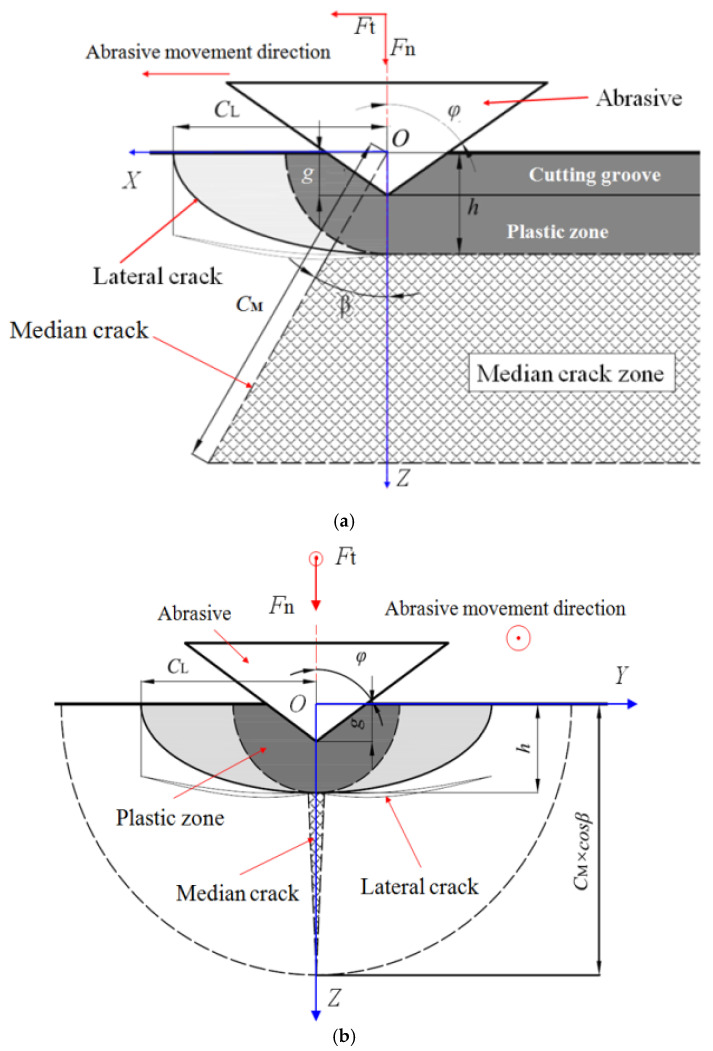
Crack system of abrasive cutting: (**a**) front view; (**b**) left view.

A schematic diagram of the diamond wire saw cutting processing is shown in Figure 1. The workpiece is fed vertically towards the direction of the diamond wire, and the diamond wire moves back and forth around the guide wheels. The diamond abrasive particles fixed on its surface perform a cutting action, removing material, forming a cutting kerf, and creating a machined surface. During the wire sawing process, the abrasive particles on the wire surface cut under the combined action of tangential and normal forces, which is similar to the process of moving the indenter to scratch. When an abrasive cut is made to remove the ingot’s surface material, the formation of a crack system below the abrasive is mainly due to the combined effect of tensile and shear stresses formed inside the material by abrasive cutting. When the abrasive achieves material brittle removal during cutting, lateral cracks initiate below the abrasive at the bottom of the plastic deformation zone caused by the abrasive pressure. After abrasive cutting, lateral cracks extend towards the material surface and eventually reach the surface to form material removal. At the same time, a median crack propagates towards the interior of the material at the bottom of the plastic deformation zone below the abrasive, forming a microcrack damage layer on the processed subsurface. In the theory of indentation mechanics, when analyzing the crack system below the indenter, the indenter is only subjected to normal forces. In the actual machining process, the cutting action of abrasive particles is the process of material fracture removal under the combined action of normal and tangential loads. In this case, there will still be a half-penny median crack below the abrasive particles, which is very similar to when the normal load acts alone. However, the length and direction of the crack will be affected by the tangential load, and the median crack will be deflected at an angle from the original vertical direction to the direction of the tangential load. This is called crack deflection angle *β*, as shown in Figure 2.

The outside of the plastic deformation zone of the material below the abrasive is the elastic deformation zone, and the initiation positions of the median and lateral cracks are at the intersection of the two zones directly below the abrasive. Therefore, lateral cracks are generated at the bottom of the plastic zone, and the depth *h* of the plastic zone is also the depth of lateral crack generation. Based on this, the expression for the depth of lateral cracks is as follows [20]:

(1) $$h=0.43{\left(\sin\varphi \right)}^{1/2}{\left(\cot\varphi \right)}^{1/3}{\left(\frac{E}{H}\right)}^{1/2}{\left(\frac{F_{n}}{H}\right)}^{1/2}$$

where *h* is the depth of lateral crack generation, μm; *E* is the elastic modulus, GPa; *H* is the processed materials hardness, GPa; *F*_n_ is the normal load on a single abrasive, N; and *φ* is the sharpness angle of the abrasive cutting edge, °.

When abrasives are processed on the material surface, the median crack extends to its maximum length when the maximum plastic deformation below the abrasive occurs during the external force loading process. The propagation of median cracks is driven by the elastic stress field and residual stress field below the abrasive. Due to the irreversibility of crack propagation, the median crack will not heal during the unloading process after abrasive cutting. When only considering the normal load, the *C*_MI_ expression for the median crack length is as follows [21]:

(2) $$C_{\mathrm{MI}}={\left[\frac{X_{e}^{M}}{X_{r}^{M}}\alpha_{K}{\left(\frac{E}{H}\right)}^{1/2}{\left(\cot\varphi \right)}^{2/3}\frac{F_{n}}{K_{\mathrm{IC}}}+\alpha_{K}{\left(\frac{E}{H}\right)}^{1/2}{\left(\cot\varphi \right)}^{2/3}\frac{F_{n}}{K_{\mathrm{IC}}}\right]}^{2/3}$$

where *α*_K_ is a dimensionless constant, *α*_K_ = 0.0366; *K*_Ic_ is the static fracture toughness of the processed material, MPa·m^1/2^; and $X_{e}^{M}$ = 0.032 and $X_{r}^{M}$ = 0.026 are the indentation coefficients for the elastic stress field and residual stress field, respectively.

In the square brackets of Equation (2), the parts preceding and following the plus sign represent the contributions of the elastic stress field under normal loading and the local residual stress field caused by the plastic zone to the median crack propagation length, respectively.

When the abrasive particles perform a cutting action, they are not only subjected to normal loads but also to tangential loads. Therefore, the propagation angle of the median crack deviates from the abrasive cutting direction, and the propagation direction and length of the median crack change. Li et al. [9] found that the median crack deflection angle *β* related to the sharpness angle *φ* of the abrasive cutting edge through analysis of the stress field distribution characteristics below the abrasives in processing silicon crystals. When *φ* is within the range of [55°, 75°], the relationship is as follows:

(3) β=−1.351φ+112.6

The participation of tangential load will not affect the plastic deformation zone depth below the abrasive, so it will not cause changes to the residual stress field generated by material plastic deformation below the abrasive, but it will cause changes in the elastic stress field. Moreover, the dynamic fracture toughness *K*_ID_ of brittle materials during processing should be considered to be approximately 30% of the static fracture toughness *K*_IC_. Therefore, the propagation length *C*_M_ of the median crack below the abrasive under the combined action of normal and tangential loads has been comprehensively considered, and its expression revised to:

(4) $$C_{M}={\left[\frac{X_{e}^{M}}{X_{r}^{M}}\alpha_{K}{\left(\frac{E}{H}\right)}^{1/2}{\left(\cot\theta \right)}^{2/3}\frac{\varepsilon F_{n}}{0.3K_{\mathrm{IC}}}+\alpha_{K}{\left(\frac{E}{H}\right)}^{1/2}{\left(\cot\theta \right)}^{2/3}\frac{F_{n}}{0.3K_{\mathrm{IC}}}\right]}^{2/3}$$

where *ε* is a correction factor considering the ratio *K* of tangential load to normal load [22], *ε* = 0.225 *K*^2^ + 0.175 *K* + 1.

## 3. Relationship between As-Sawn SR and SSD

**Figure 3:**
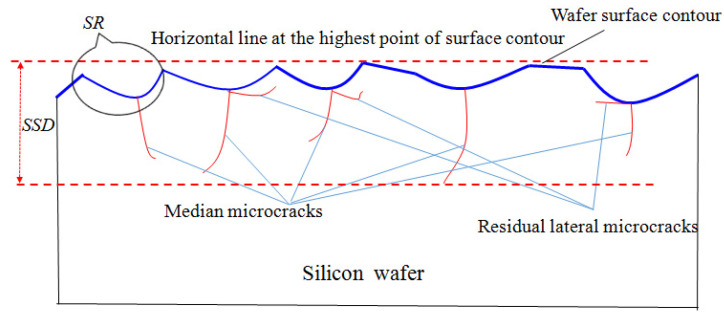
Schematic diagram of generated surface profile and subsurface microcrack damage caused by material brittleness removal in sawing.

When a diamond wire saw is used to process mono-Si, brittle pits are formed on the processed surface when the material is removed in a brittle mode, resulting in surface roughness. The propagation depth of residual median cracks in the subsurface is the depth of the crack damage layer. A schematic diagram of the generated surface profile and subsurface microcrack damage is shown in Figure 3.

The surface of the as-sawn wafer presents a peak-to-valley contour, which conforms to the definition of the surface roughness value *R*_z_. Its value is approximately equal to the distance from the bottom of the plastic zone after abrasive cutting, in which lateral cracks are generated to the highest point of the surface contour, that is, the surface roughness *R*_z_ value is approximately equal to the lateral crack generation depth *h*:

(5) $$\mathrm{SR}\;(R_{z})\;\approx \;h=0.43{\left(\sin\varphi \right)}^{1/2}{\left(\cot\varphi \right)}^{1/3}{\left(\frac{E}{H}\right)}^{1/2}{\left(\frac{F_{n}}{H}\right)}^{1/2}$$

The SSD of the as-sawn silicon wafer is taken as the longest propagation depth of the median crack, defined as the distance from the bottom of the longest median crack to the highest point of the silicon wafer surface contour, as shown in Figure 3. Therefore,

(6) $$\mathrm{SSD}=C_{M}\cos\beta$$

as follows:

(7) $$\mathrm{SSD}={\left[\frac{X_{e}^{M}}{X_{r}^{M}}\alpha_{K}{\left(\frac{E}{H}\right)}^{1/2}{\left(\cot\varphi \right)}^{2/3}\frac{\varepsilon F_{n}}{0.3K_{\mathrm{IC}}}+\alpha_{K}{\left(\frac{E}{H}\right)}^{1/2}{\left(\cot\varphi \right)}^{2/3}\frac{F_{n}}{0.3K_{\mathrm{IC}}}\right]}^{2/3}\cdot \cos\beta$$

Equations (5) and (7) both contain the normal load *F*_n_ on the abrasive. By combining these two equations to eliminate the normal load *F*_n_, the relationship between the SSD and the SR *R*_z_ is constructed as follows:

(8) $$\mathrm{SSD}=3.08{\left(1+\frac{X_{e}^{M}}{X_{r}^{M}}\varepsilon \right)}^{2/3}\alpha_{K}^{2/3}\frac{1}{{\left(\sin\varphi \right)}^{2/3}}\frac{H}{{\left(0.3K_{\mathrm{IC}}\right)}^{2/3}E^{1/3}}\cdot \cos\beta \cdot {\mathrm{SR}}^{4/3}$$

From Equation (8), it can be seen that when the processing state and abrasive parameters are determined, the SSD of the silicon wafer exhibits a non-linear relationship with the SR *R*_z_. According to Equation (8), by measuring the SR of the as-sawn silicon wafer, the SSD of the silicon wafer can be calculated and predicted.

## 4. Sawing Experiment

The sawing experiment was conducted using a reciprocating single-wire diamond wire saw cutting machine which was made by Shenyang Kejing Co., Ltd., Shenyang, China. The sawing process parameters used are shown in Table 1. Six sets of sawing experiments were completed using two wire speeds and three workpiece feed speeds. The size of the sawn mono-Si was 10 mm × 10 mm × 40 mm, which was cut along the (111) crystal plane, and the wafer thickness was 0.5 mm. The diameter of the diamond core wire used was 120 μm, the diamond abrasive particle size was 15–20 μm, and the effective cutting length of the diamond wire was 20 m.

**Figure 4:**
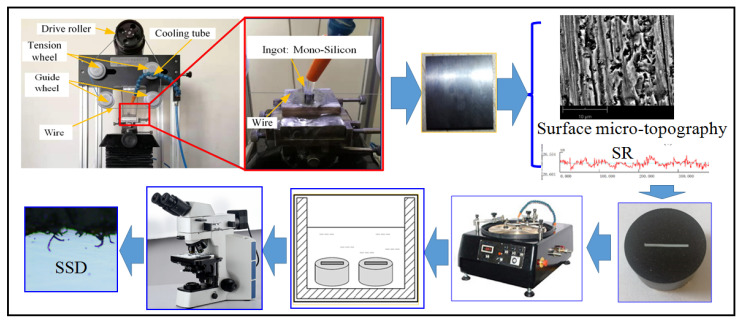
Experimental process.

After ultrasonic cleaning of the silicon wafers, the surface morphology was observed using a scanning electron microscope (Hitachi, Japan). A contact surface roughness measuring instrument was used to select five points in the middle of the wafer surface perpendicular to the wire mark to measure the surface roughness *R*_a_, and the average of the measurement values was taken. Subsequently, the silicon wafer was embedded, and the thick section of the wafer perpendicular to the direction of diamond wire movement was ground and polished, and then corroded in a hydrofluoric acid solution for 15 s. The specimen was cleaned with distilled water and the SSD was observed using an OLYMPUS optical microscope. Five locations were taken from each group of specimens for observation and statistics, and the average of the measured SSD values was taken. The relationship between as-sawn silicon wafer SR and SSD was analyzed. The experimental steps are shown in Figure 4.

## 5. Results and Discussion

### 5.1. Surface Micromorphology and SSD of As-Sawn Cut Silicon Wafers

**Figure 5:**
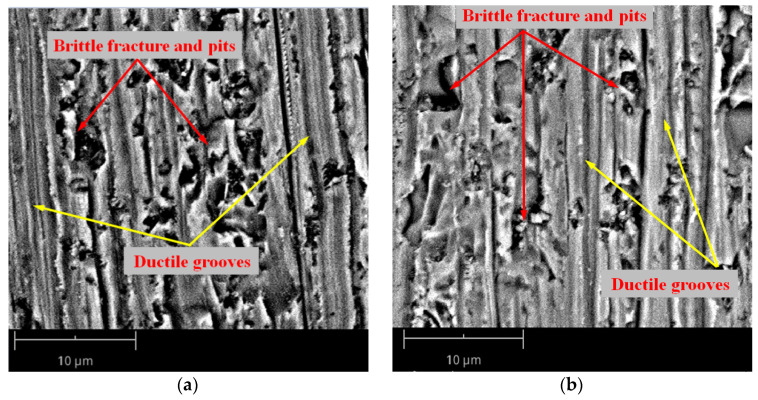
Micromorphology of as-sawn silicon wafer surface: (**a**) *V*_s_ = 48 m/min, *V*_w_ = 0.54 mm/min; (**b**) *V*_s_ =78 m/min, *V*_w_ = 0.18 mm/min.

Figure 5 shows the surface micromorphology of the silicon wafer observed using scanning electron microscopy with cutting parameters of *V*_s_ = 48 m/min, *V*_w_ = 0.54 mm/min, *V*_s_ = 78 m/min, and *V*_w_ = 0.18 mm/min. The former group of parameters adopted a low wire speed and a relatively high feed speed, while the latter group of parameters adopted a relatively high wire speed and a relatively low feed speed. The surface of the silicon wafer shown in Figure 5a is mainly composed of brittle fractures and pits formed by abrasive brittle material removal, presenting a large area of fragmented morphology. At the same time, there are still a small number of plastic grooves formed by material being removed by the ductile shearing of the abrasive particles, which will not form microcrack damage in the subsurface layer. There are still a large number of brittle fractures and pits in the microstructure of the silicon wafer surface shown in Figure 5b, but their proportion has decreased compared to Figure 5a.

**Figure 6:**
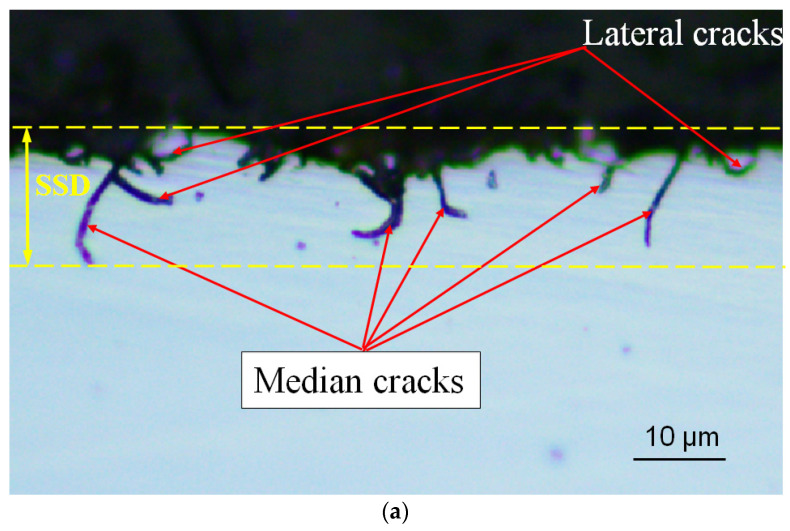

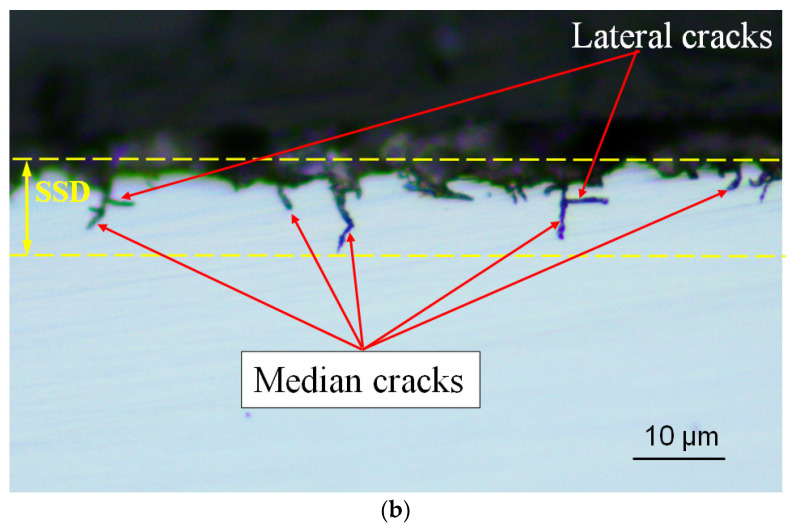
Subsurface microcracks of as-sawn silicon wafer: (**a**) *V*_s_ = 48 m/min, *V*_w_ = 0.54 mm/min; (**b**) *V*_s_ = 78 m/min, *V*_w_ = 0.18 mm/min.

The subsurface microcrack damage morphology of the silicon wafers can be observed, as shown in Figure 6. The presence of median cracks in the subsurface layer of the sawn silicon wafers can be clearly observed. In addition to the median cracks that propagate into the interior of the material, there are also a small number of lateral cracks remaining in the subsurface of the silicon wafer that have not been extended to remove material. Most lateral crack propagation achieves material removal; therefore, the observed number of lateral cracks is lower than the number of median cracks. The measured SSD of the as-sawn silicon wafer using the process parameters used in this paper is about 12–16 μm. Comparing Figure 6a,b, it can be observed that as the wire speed increases and the workpiece feed speed decreases, the propagation length of the median crack in the subsurface layer of the silicon wafer decreases, which is consistent with the research findings of other scholars.

### 5.2. Prediction of SSD of As-Sawn Cut Silicon Wafers

In the third section of this paper, the relationship between SR and SSD was constructed. According to Equation (8), the SSD of silicon wafers can be calculated and predicted by measuring the SR *R*_z_ value. When using Equation (8) to calculate and predict the SSD in diamond wire sawing of mono-Si, it is necessary to determine the values of each factor in the equation. When sawing the (111) crystal plane of mono-Si, the material characteristic parameters are *E* = 187 GPa, *H* = 9 GPa (Mohs hardness), and *K*_IC_ = 0.82 MPa·m^1/2^. The surface roughness *R*_z_ generally needs to be detected using a light-cutting microscope, which may have some human error. Therefore, the surface roughness *R*_a_ of the sawn silicon wafers is measured, and then converted based on the approximate relationship between *R*_a_ and *R*_z_. When *R*_a_ < 2.5 μm, *R*_z_ ≈ 5 *R*_a_.

Mahmoud et al. [23] conducted morphological statistics on diamond abrasives and found that the tip half angle *φ* of diamond abrasives mainly distributed within the range of [55°, 75°] and followed a normal distribution. Therefore, to simplify the calculation, the tip half angle of the diamond abrasives is taken here as *φ* = 65°. Therefore, according to Equation (3), the deflection angle of the median crack is calculated as *β* = 24.785°. In Equation (8), *ε* is a correction factor considering the ratio value *K* of tangential load to normal load, and *ε* = 0.225 *K*^2^ + 0.175 *K* + 1. During abrasive cutting, the normal and tangential loads acting on the abrasive can be calculated by multiplying the projected area of contact between the abrasive and the material in the force direction by the material hardness. Li et al. [9] derived the expression for the tangential load to normal load ratio *K* during abrasive cutting, as shown in Equation (9). According to this equation, it is calculated that *ε* = 1.072.

(9) $$K=\frac{2}{\pi \cdot \tan\varphi }$$

Based on the above-determined parameter values, the *R*_a_ of as-sawn silicon wafers can be measured and substituted into Equation (8) to calculate the SSD.

### 5.3. Experimental Results and Theoretical Model Validation

The experimental measurement of SR and SSD, and the theoretical predictions of the SSD of as-sawn cut silicon wafers under six sets of process parameters are listed in Table 2. The relative error between predicted values and experimental measurement values is analyzed. From the experimental results, it can be seen that at the same wire speed, as the feed speed increases, the SR and SSD of the as-sawn silicon wafer also increase. At the same feed speed, the wire speed decreases, and the SR and SSD of the as-sawn silicon wafer increase.

**Figure 7:**
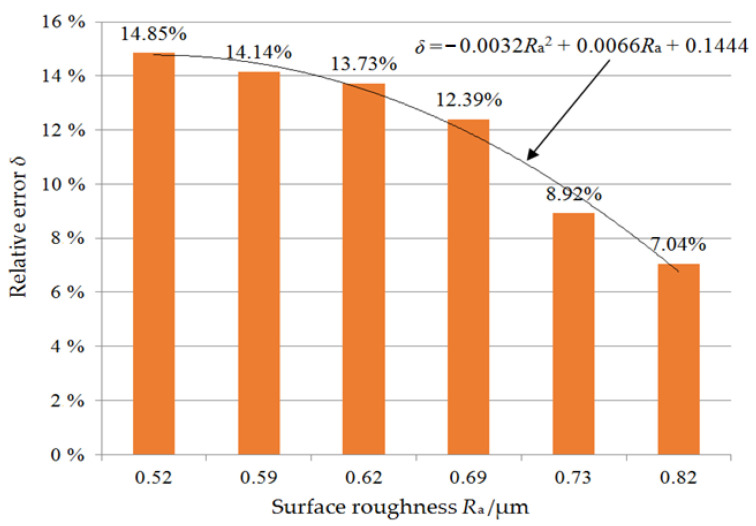
The relative error between the predicted and experimental SSD values of as-sawn silicon wafers.

Figure 7 shows the relative error of SSD of as-sawn silicon wafers between the predicted and experimentally measured values under six sets of process parameters. Overall, the theoretical predicted values are in good agreement with the experimental measurement results, with a relative error of within 15%. The fitting curve of the relative error *δ* with SR *R*_a_ conforms to a quadratic function relationship. It can be seen that the larger the SR value of the as-sawn silicon wafer, the lower the relative error of the SSD, that is, the closer the theoretical predicted value is to the experimental measurement value. Based on the surface morphology of the as-sawn silicon wafer shown in Figure 5, it can be seen that the proportion of ductile domain removal increases in the overall material removal when using relatively high sawing speed and low feed speed. The SSD prediction model in this paper is based on the fracture mechanics theory of material brittle removal and is not suitable for material ductile removal during processing. The material removed in a ductile manner will form shallower grooves than brittle pits. When measuring SR in randomly selected areas, the SR value generated by the material ductile removal is relatively low. The theoretical prediction value of SSD using Equation (8) also decreases. However, the actual SSD still depends on the maximum value of median crack propagation caused by the abrasive brittle removal of the material. Therefore, in this case, the error of SSD between the predicted value and the experimental value increases. This prediction model is more suitable for predicting the SSD of the as-sawn subsurface during material brittle removal. In current industrial production, the cutting process of silicon crystals requires speed, that is, using high feed speed to improve sawing efficiency, and the surface material is usually removed in a brittle manner. This means that the SSD evaluation model established in this paper is suitable for industrial production applications of wire saw slicing mono-Si wafers.

According to the determination of various parameters in Equation (8), it can be inferred that SSD = 19.254 *R*_a_^4/3^, indicating a non-linear increasing relationship between SSD and SR. The larger the SR value, the deeper the SSD. From the comparison results of SSD of the as-sawn silicon wafers shown in Table 2, it can be seen that the predicted values are all slightly lower than the experimental measurements. The reasons for this are identified when establishing that the relationship between as-sawn silicon wafer SSD and SR is based on the indentation mechanics theory of a single abrasive and the stress field distribution of a single abrasive scratch. However, in actual sawing processing, the removal of material is the result of multiple abrasives. The peak of the surface contour formed by lateral crack propagation below one diamond abrasive after removing the material may be removed by lateral crack propagation below another adjacent abrasive. That is to say, the peak-valley value of the actual contour formed on the as-sawn surface of the silicon wafer is lower than the theoretical contour peak-valley value formed based on a single abrasive brittle removal material. On the other hand, in the actual sawing process, the cutting effect of one abrasive will also strengthen the stress field below the adjacent abrasive, increasing the median crack propagation length and causing the actual crack damage depth to be greater than the theoretical calculation value.

**Figure 8:**
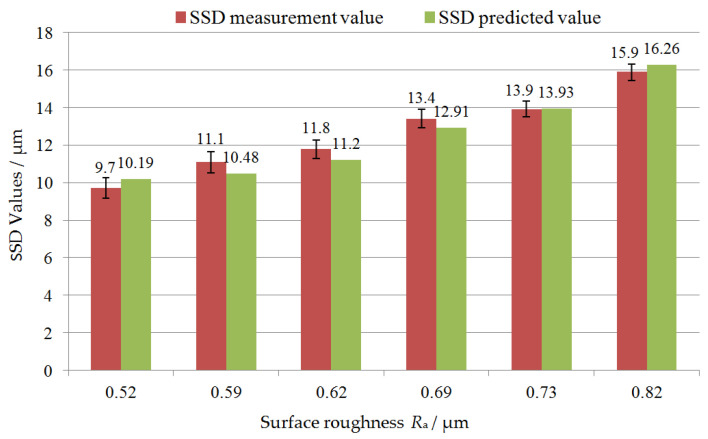
Comparison between the improved predicted values and experimental measurements of SSD, and the relationship between SSD and surface roughness *R*_a_.

Based on the analysis of the relative error between the predicted and measured values, the equation for SSD = 19.254 *R*_a_^4/3^ can be modified, and the coefficient *η* to characterize the influence of material ductile removal on *R*_a_ value was added, namely, SSD = *η*·19.254 *R*_a_^4/3^ and *η* = 1.1, and the improved relationship is SSD = 21.179 *R*_a_^4/3^. In this way, the predicted SSD values based on the theoretical model are closer to the experimental measurement values. Figure 8 shows the comparison of the as-sawn silicon wafer SSD between the predicted values calculated by adopting the improved theoretical model and experimental measurements, and shows the corresponding relationship between SSD and SR *R*_a_. The theoretical prediction value of SSD calculated after model improvement is very close to the experimental detection value, and the relative error between the two is reduced to within 5%. Based on the established theoretical prediction model, the SSD of silicon wafers can be quickly and non-destructively predicted by measuring the SR of the as-sawn surface, which can provide guidance for optimizing the sawing process.

## 6. Conclusions

The relationship between the SSD and the SR of the as-sawn silicon wafers has been established in this paper. Furthermore, the prediction model for evaluating the SSD of mono-Si sawn along the (111) crystal plane has been verified and improved through experiments. The following conclusions have been drawn:

(1) A decrease in wire speed and an increase in feed speed result in an increase in SR and SSD of silicon wafers.
(2) When cutting mono-Si with a diamond wire saw, there is a non-linear increasing relationship between the SSD and the SR of the silicon wafer, and the larger the SR value, the deeper the SSD.
(3) The theoretical prediction value of SSD is in good agreement with the experimental measurement results. The larger the SR value of the as-sawn silicon wafer, the smaller the relative error of the SSD. The relationship between SSD and *R*_a_ has been further refined by adding a coefficient considering the influence of material ductile regime removal on *R*_a_ values when cutting mono-Si along the (111) crystal plane, so the improved relationship is SSD = 21.179 *R*_a_^4/3^. Then the predicted SSD value is closer to the experimental measurement value.

Based on the prediction model that has been established in this paper, the SSD of as-sawn silicon wafers along the (111) crystal plane can be quickly and non-destructively predicted, which can provide an effective reference for optimizing the diamond wire saw cutting silicon wafer process.

## Figures and Tables

**Table 1:** Processing Parameters.

No.	Wire Speed vs. (m/min)	Feed Speed *V*_w_ (mm/min)
1	78	0.18
2	78	0.36
3	78	0.54
4	48	0.18
5	48	0.36
6	48	0.54

**Table 2:** Comparison of SSD between predicted and experimental measured values.

No.	Wire Speed *V*_s_ (m/min)	Feed Speed *V*_w_ (mm/min)	SR *R*_a_ (μm)	SSD (μm)		Relative Error between Predicted and Experimental Values
				Measured Values	Predicted Values	(%)
1	78	0.18	0.53	9.7	8.26	14.85%
2	78	0.36	0.62	11.8	10.18	13.73%
3	78	0.54	0.69	13.4	11.74	12.39%
4	48	0.18	0.59	11.1	9.53	14.14%
5	48	0.36	0.73	13.9	12.66	8.92%
6	48	0.54	0.82	15.9	14.78	7.04%

## Data Availability

Data are contained within the article.
